# Clinical and Ultrasound Biomicroscopic Characteristics of Congenital Fibrovascular Pupillary Membrane-Induced Secondary Glaucoma

**DOI:** 10.3389/fmed.2021.763137

**Published:** 2021-10-28

**Authors:** Yingting Zhu, Lei Fang, Yimin Zhong, Julius Oatts, Ying Han, Shufen Lin, Liming Chen, Xiaodi Zhou, Yihua Su, Pingping Liu, Xing Liu

**Affiliations:** ^1^State Key Laboratory of Ophthalmology, Zhongshan Ophthalmic Center, Sun Yat-sen University, Guangzhou, China; ^2^Department of Ophthalmology, School of Medicine, University of California, San Francisco, San Francisco, CA, United States

**Keywords:** congenital fibrovascular pupillary membrane, secondary glaucoma, ultrasound biomicroscopy, clinical characteristics, classification

## Abstract

**Purpose:** The purpose of this study was to describe and summarize the clinical features of congenital fibrovascular pupillary membrane-induced secondary glaucoma (CFPMSG).

**Design:** Cross-sectional case series.

**Methods:** Eyes of 32 patients with CFPMSG were enrolled. Demographic data, including gender, laterality, age at presentation, and age at onset of glaucoma were collected. Patients underwent comprehensive ophthalmic examinations and ultrasound biomicroscopy (UBM). CFPMSG eyes were classified into three groups based on UBM findings and intergroup analysis was performed using ANOVA.

**Results:** The average age at presentation was 2.4 ± 4.6 months (mean ± SD) and at glaucoma onset was 3.8 ± 4.5 months. Compared to normal fellow eyes, all affected eyes had increased intraocular pressure (IOP), axial length, corneal diameter, and central corneal thickness, and decreased anterior chamber depth (ACD) (all *P* ≤ 0.001). Twenty-two affected eyes (68.8%) had evidence of glaucomatous optic neuropathy. Based on iris configuration on UBM, eyes were classified as 53% type I (“U” shape), 34% type II (“Y” shape), and 13% type III (no anterior chamber). IOP in types II (33.8 ± 5.9 mmHg) and III (35.2 ± 5.9 mmHg) was significantly higher than in type I eyes (26.5 ± 5.1 mmHg). The ACD was shallower in type II compared to type I (*P* = 0.045).

**Conclusion:** Congenital fibrovascular pupillary membrane-induced secondary glaucoma is characterized by ocular hypertension, corneal enlargement and edema, axial length elongation, and glaucomatous optic neuropathy. Glaucoma in this condition is secondary to pupillary block and angle-closure. UBM provides important information for the diagnosis and classification of CFPMSG. This novel classification system demonstrated varying levels of severity and may guide on management of this disease.

## Introduction

Congenital fibrovascular pupillary membrane (CFPM) refers to a condition where a congenital white membrane covers part or all of the pupil and may be accompanied by anterior chamber angle dysplasia (1). It is most commonly unilateral. It was originally believed to be the result of abnormal migration and differentiation of anterior chamber neural crest cells, but a recent histopathological study suggests that it is a variant of persistent fetal vasculature (2, 3). If the membrane blocks the pupil completely, iris bombé and anterior chamber angle closure may occur. Additionally, children with CFPM may demonstrate progressive occlusion of the pupil leading to deprivational amblyopia or/and increased intraocular pressure (IOP) leading to optic nerve damage and irreversible vision loss (4).

Most cases of CFPM are sporadic. The first reported cases in 1986 were called congenital pupil-iris-lens membrane with goniodysgenesis (1). Subsequent cases have been described (2–7), though without detailed imaging of the anterior chamber. More recently, another group described the clinical characteristics of 13 cases of CFPM, though reported unsatisfactory surgical results with a high recurrence rate and poor surgical success (8). This emphasizes the importance of better understanding and classifying this condition to improve visual outcomes.

While the slit-lamp examination is useful in visualizing pupillary membranes, ultrasound biomicroscopy (UBM), a widely used anterior segment modality using ultrahigh-frequency sound, can provide a more detailed and complete view of the anterior segment pathology. To our knowledge, this is the first study to use UBM to image and classify CFPM. This novel classification system may provide better insight into the pathophysiology of congenital fibrovascular pupillary membrane-induced secondary glaucoma (CFPMSG).

## Methods

### Patients

This was a retrospective cross-sectional study of all children with CFPMSG seen at the Zhongshan Ophthalmic Center, Sun Yat-sen University, between 2013 and 2020. This study was approved by the Zhongshan Ophthalmic Center Institution Review and Ethics Board (2020KYPJ121). All parents of the patients provided informed consent following the Declaration of Helsinki. Inclusion criteria were (1) age <16 years, (2) unilateral presentation, (3) presence of a fibrovascular membrane attached to the iris stroma adjacent to the pupil, and (4) initial IOP >21 mmHg. Exclusion criteria included patients with bilateral disease or other types of glaucoma. Demographic data were collected, including gender, age at referral to our clinic, age at presentation, age at onset of glaucoma, eye laterality, and parental chief complaint.

### Ophthalmic Examination

Examinations under anesthesia were performed using chloral hydrate 10% and topical anesthesia. All patients underwent hand-held slit-lamp biomicroscopy (Keeler, Bucks, England) and slit-lamp photography (BX900; Haag-Streit AG, Koniz, Switzerland). Corneal opacity was classified as mild (iris details clearly visible), moderate (iris details partially visible), or severe (no iris details visible) (9). Horizontal corneal diameter (HCD) was defined as the white-to-white distance from 3 to 9 o'clock, measured using a caliper. IOP was measured using a Tono-pen Avia (Reichert, Depew, New York, USA). A- and B-scan ultrasonography were performed to measure the axial length (AL) and evaluate the posterior segment of the eye (Quantel Medical, CF, France; Nidek US-1800, Japan). The cross-section image of the optic nerve head was evaluated. It was considered glaucomatous cupping when we observed a depression anterior to the optic nerve, which is limited to the papilla (10). All ultrasound examinations were carried out by a single experienced examiner (PPL).

### Ultrasound Biomicroscopy

UBM was performed under anesthesia with 50 MHz resolution and the patient was in the supine position (model SW-3200L; Tianjin Suowei Electronic Technology Co, Ltd., Tianjin, China). Saline was used as the coupling agent, and the probe was always perpendicular to the ocular surface. UBM images of the anterior segment were obtained under standard room illumination with axial and radial scans at the 3, 6, 9, and 12 o'clock positions. We selected high-quality images from each o'clock's position and one image of the central anterior chamber to measure anterior chamber parameters and estimation of pupil position. Central corneal thickness (CCT) was measured on UBM in the center of the cornea from the inner surface of the corneal endothelium to the outer epithelial surface. Anterior chamber depth (ACD) was measured as the axial distance from the corneal endothelium to the anterior lens surface. If the anterior lens surface was not visible due to the membrane, the most posterior point of the iris synechiae in front of the lens was used as the reference point. Iris configuration was recorded and classified. All UBM examinations were carried out by a single experienced examiner (LMC).

### Statistical Analysis

Descriptive statistics were reported as means and SD, medians and ranges, or numbers and percentages as appropriate. The normality of the continuous variable distribution was examined using the Kolmogorov–Smirnov test. For comparison between affected and unaffected eyes, Student's paired *t*-test or Kruskal–Wallis *H*-test was used according to whether variables conformed to a normal distribution. The ANOVA or Pearson's chi-squared test and Fisher's exact test were used for comparison between UBM-defined CFPMSG subtypes. Bonferroni correction was used for multiple comparisons, statistical significance was defined as *P* < 0.05. All statistical analysis was performed using SPSS software, version 22.0 (SPSS, Inc., Chicago, IL, USA).

## Results

### Demographics

In total, 32 eyes of 32 patients with CFPMSG were included in this study, and demographic data are summarized in Table 1. Seventeen patients were noted to have an abnormality by their parents (such as white pupil) at birth, and four (24%) presented with symptoms associated with glaucoma (cloudy cornea, corneal enlargement, epiphora, photophobia, ocular redness, eye pain, and vomiting) within 1 month of age. The remaining 15 patients had no symptoms at birth, and 12 of them (80%) were first noticed and presented with glaucomatous symptoms at a median age of 3 months. All patients but one were born at full term. Two of the children were twins with unaffected twin siblings. Systemic conditions included G6PD deficiency (2) and congenital heart disease (1). No other abnormalities, including facial, dental, or umbilical malformations, were reported. There was no family history of pupillary abnormalities or glaucoma. All of the subjects were negative in serological tests for rubella.

**Table 1:** Demographic data of patients with congenital fibrous pupillary membrane-induced secondary glaucoma.

**Variables**	
**Gender, n (%)**	
Male	17 (53.1%)
**Laterality, n (%)**	
Right	18 (56.3%)
**Age at first presentation, months**	
Median (range)	0 (0–24)
**Age at onset of glaucoma, months**	
Median (range)	3.0 (0–24)
**Age at referral, months**	
Median (range)	4.3 (1–24)
**Chief complaint, n (%)**	
Enlarged or cloudy cornea	15 (46.9%)
White pupil	11 (34.4%)
Epiphora and photophobia	3 (9.4%)
Ocular redness and pain	2 (6.3%)
Vomiting	1 (3.1%)

### Ophthalmic Examination

A comparison of clinical data between affected and unaffected eyes is shown in Table 2. IOP, AL, HCD, and CCT were all significantly higher in affected eyes compared to normal fellow eyes (*P* < 0.001). Mean ACD was 1.50 ± 0.99 mm in affected eyes compared to 2.19 ± 0.33 mm in unaffected eyes (*P* = 0.001). All patients presented with IOP > 21 mmHg. Seven patients presented with HCD <12 mm with a median age at the time of referral of 9 months (range 1–24 months), which is significantly older than the rest of patients with the median age at the referral of whom of 4 months (range 1–10 months) (*P* = 0.033). Moreover, based on the AL-to-age data of Sampaolesi and Caruso (11), we found that four of seven patients (57.1%) with HCD <12 mm had initial AL within normal range, while four of 25 patients (16%) with HCD ≥ 12 mm did. All patients had a white membrane covering the pupil and iris bombé with peripheral anterior synechiae. Most patients (87.5%) had pupil displacement. B-scan ultrasound showed optic nerve cupping in 22 eyes (68.8%).

**Table 2:** Comparison between affected and fellow eyes in subjects with congenital fibrous pupillary membrane-induced secondary glaucoma at the time of referral.

**Variables**	**Affected eye**	**Fellow eye**	***P*** -value^*^
**IOP, mmHg**			
Mean (SD)	30.1(6.6)	14.6(3.0)	**<0.001**
Range	17.3–43.4	8.5–20.6	
**HCD, mm**			
Mean (SD)	12.3 (0.9)	10.8(0.4)	**<0.001**
Range	10.5–14.0	10–11.5	
**AL, mm**			
Mean (SD)	22.7(1.8)	19.3(1.5)	**<0.001**
Range	19.5–26.6	16.6–21.8	
**CCT**, **μm**			
Mean (SD)	629.3(110.7)	528.3(34.9)	**<0.001**
Range	450.0–920.0	460.0–580.0	
**ACD, mm**			
Mean (SD)	1.50(0.99)	2.19(0.33)	**0.001**
Range	0–3.92	1.62–2.70	
**Corneal opacity, n (%)**			
Mild	23 (71.9%)	–	
Moderate	9 (28.1%)	–	
Severe	0(0%)		
**Corectopia, n (%)**			
None	4 (12.5%)	–	
Superior	3 (9.4%)	–	
Nasal	11 (34.4%)	–	
Temporal	14 (43.8%)	–	

*ACD, anterior chamber depth; AL, axial length; CCT, central corneal thickness; HCD, horizontal corneal diameter; IOP, intraocular pressure; mmHg, millimeters of mercury.*

*Independent samples t-tests were used for continuous variables.*

* *Comparison between fellow eye and affected eye.*

*P-values with statistical significance (<0.05) are highlighted in bold*.

### Ultrasound Biomicroscopy Classification

**Figure 1:**
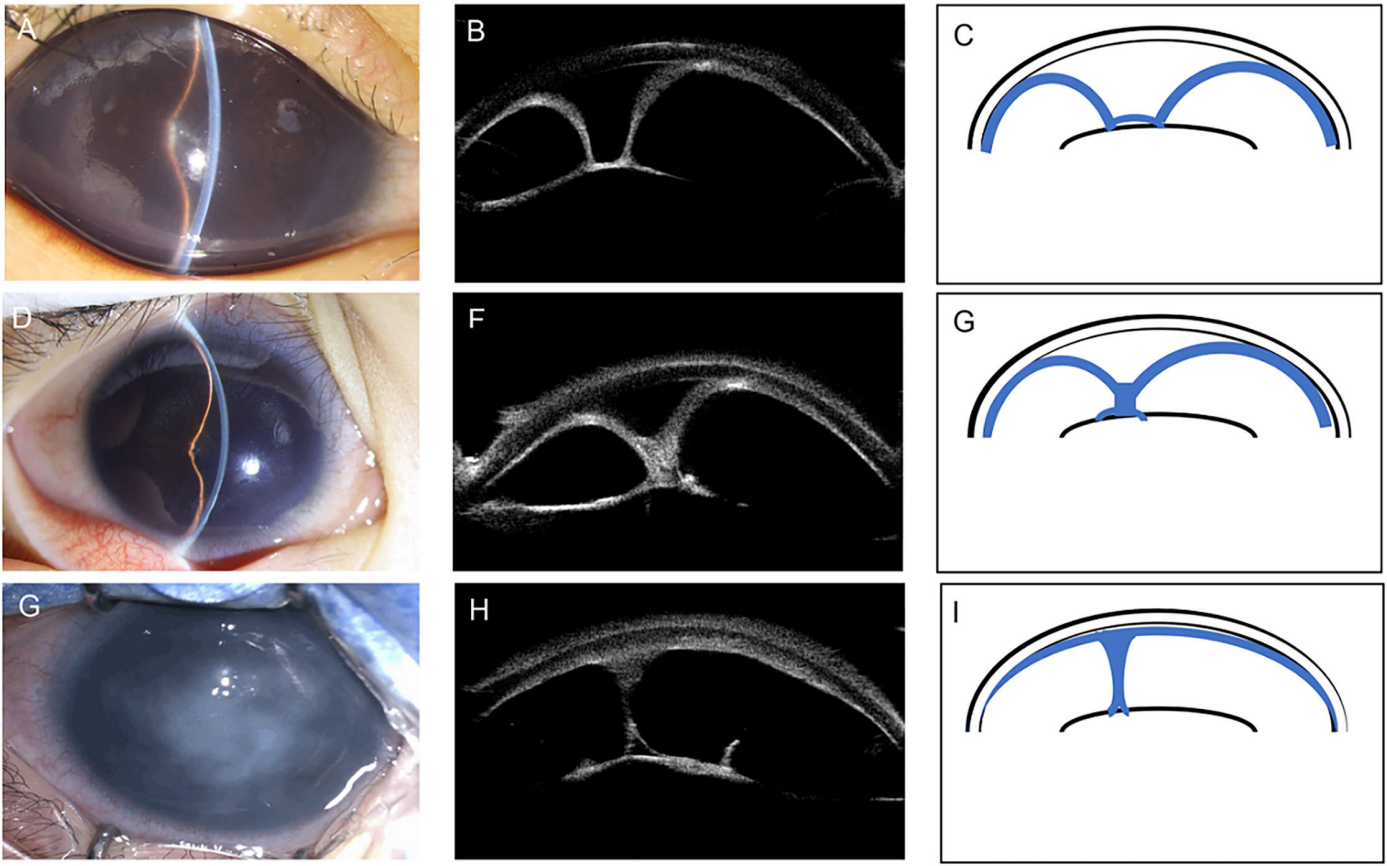
Anterior segment photography, corresponding ultrasound biomicroscopy (UBM) images, and schematic illustrations demonstrating the three different types of congenital fibrovascular pupillary membrane-induced secondary glaucoma (CFPMSG). **(A–C)** Type I CFPMSG: “U”-shape configuration of iris, with complete pupillary obstruction with a fibrovascular membrane and iris bombé with peripheral anterior synechiae, but some central anterior chamber depth. **(D–F)** Type II CFPMSG: “Y”-shape configuration of iris with more severe iris bombé and peripheral anterior synechiae and central iris adhesion to the fibrotic membrane. **(G–I)** Type III CFPMSG: absent anterior chamber depth detectable, manifested as complete iris-cornea contact.

Based on the iris configuration and ACD, we classified CFPMSG into three types (Figure 1, Table 3).

**Table 3:** Comparison between different configurations of congenital fibrous pupillary membrane-induced secondary glaucoma.

**Variables**	**Type I, “U” shape**	**Type II, “Y” shape**	**Type III, absent AC**	***P*** **-values**
	**(*n* = 17)**	**(*n* = 11)**	**(*n =* 4)**	
**Age at first presentation, months**				
Median (range)	0 (0–24.0)	0 (0–7.0)	1.3 (0–5.0)	0.685
**Age at glaucoma onset, months**				
Median (range)	2.0 (0–24.0)	3.0 (0–7.0)	3.5 (1.5–7.0)	0.972
**Age at referral, months**				
Median (range)	4.0 (1.0–24.0)	4.7 (2.5–9.0)	7.0 (2.0–8.0)	0.966
**IOP, mmHg**				
Mean (SD)	26.5(5.1)	33.8(5.9)	35.2(5.9)	**0.002^a^/0.654^b^/** **0.008^c^**
**HCD, mm**				
Mean (SD)	12.3(1.0)	12.1(0.8)	12.6(0.8)	0.63
**AL, mm**				
Mean (SD)	22.3(1.8)	23.3(1.8)	23.1(1.5)	0.35
**CCT, μm**				
Mean (SD)	628.1(79.9)	611.8(140.1)	700.0(144.2)	0.648
**ACD, mm**				
Mean (SD)	1.97(0.92)	1.34(0.62)	0	**0.045^a^/0.006^b^/ <0.001^c^**
**Optic nerve cupping, n (%)**	11(64.7%)	7(63.6%)	4(100%)	0.353

*ACD, anterior chamber depth; AL, axial length; CCT, central corneal thickness; HCD, horizontal corneal diameter; IOP, intraocular pressure; mmHg, millimeters of mercury.*

*ANOVA was used for continuous variables. The Pearson's chi-squared test and the Fisher's exact test were used for the categoric variables.*

a *Comparison between type II and type I.*

b *Comparison between type III and type II.*

c *Comparison between type III and type I.*

*P-values with statistical significance (<0.05) are highlighted in bold*.

Type I (“U” shape) was found in 17 eyes (53%). It was characterized as 360-degree iridolenticular adhesions with resultant iris bombé and peripheral iridocorneal contact. A thick and hyperreflective structure blocked the pupil, pupillary margin, pupillary membrane, and was completely adherent to the anterior surface of the lens. In this group, the median age at referral was 4 months with moderate IOP elevation.

Type II (“Y” shape) was found in 11 eyes (34%). It was characterized by more severe iris bombé with fibrotic adhesions not only between the iris and lens but also between the elevated portions of the iris blocking the pupil. The thick hyperreflective membrane in this type is larger with iris-iris adhesion. The median age at referral was 4.7 months. The IOP detected at the referral of type II eyes was significantly higher than type I eyes (*P* = 0.002) and ACD was shallower than type I (*P* = 0.045).

Type III [absent anterior chamber (AC)] was found in four eyes (13%). It was characterized by the complete absence of the anterior chamber due to total iridocorneal opposition. Some strands of the iris can be seen connected to the membrane on the anterior surface of the lens. The median age at referral was 7.0 months. Mean IOP was significantly higher than that of type I eyes (*P* = 0.008), but not type II eyes (*P* = 0.654). ACD was shallower than type I (*P* < 0.001) and type II (*P* = 0.006).

We also compared other clinical characteristics, including AL, HCD, and CCT between types and found no significant difference (*P* = 0.350, 0.614, and 0.648, respectively). There were more eyes with glaucomatous optic neuropathy in type III, though this did not achieve statistical significance (*P* = 0.353).

### Case Report

**Figure 2:**
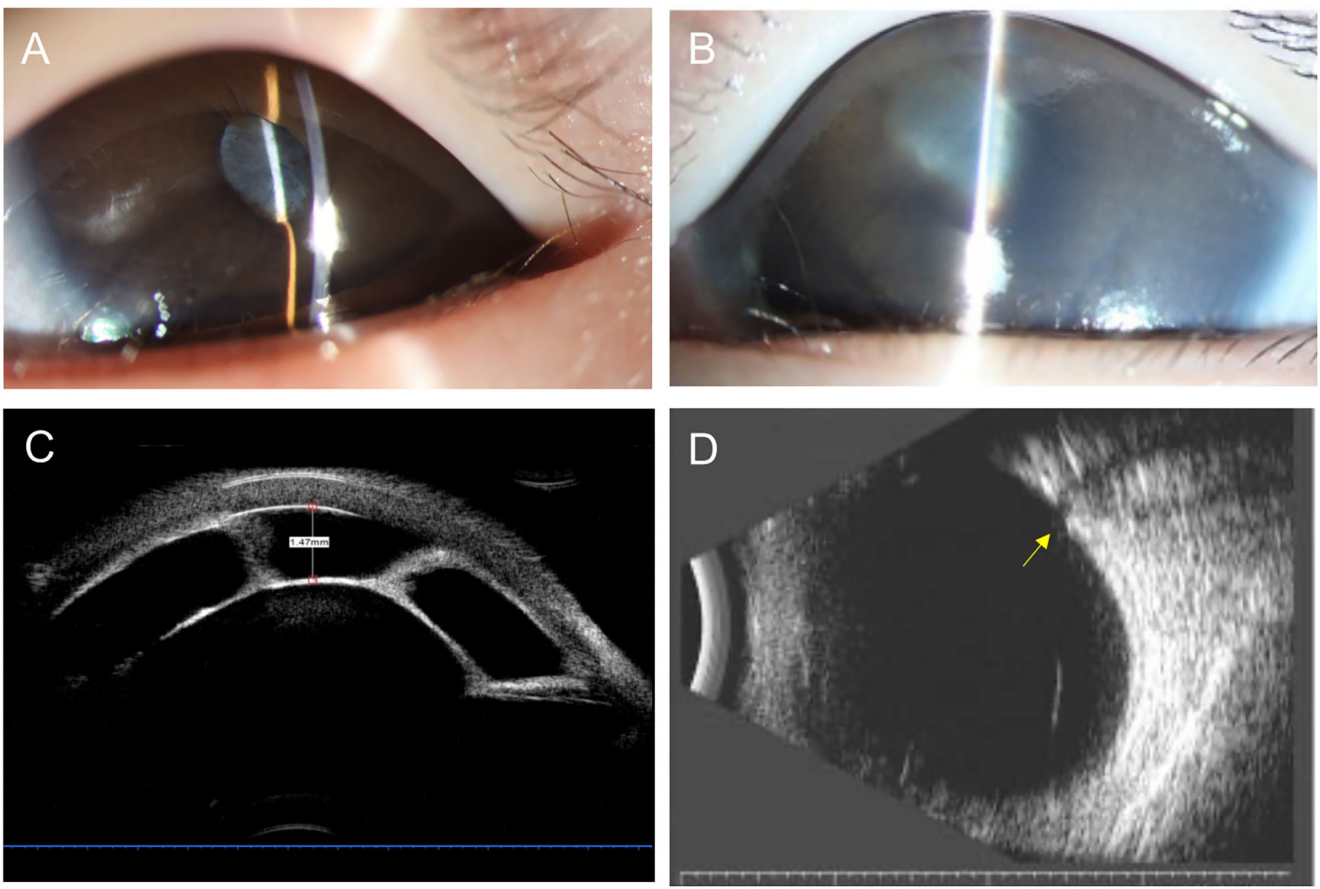
Anterior segment photography, ultrasound biomicroscopy (UBM), and B-scan ultrasonography of a 4-month-old child with congenital fibrous pupillary membrane-induced secondary glaucoma. **(A)** Anterior segment photograph at 3 months of age showing a clear cornea and normal central and peripheral anterior chamber depth. The pupil is covered by a white vascularized membrane with a few areas of posterior synechiae. **(B)** Anterior segment photograph at 4 months of age showing moderate corneal edema, iris bombé with the very shallow peripheral anterior chamber, and posterior synechiae covering 360 degrees of the pupil. **(C)** UBM demonstrates a fibrous membrane adherent to the anterior lens capsule covering the entire pupil leading to iris bombé and peripheral anterior synechiae. Central anterior depth measured 1.47 mm. **(D)** B-scan ultrasound image demonstrates significant cupping of the optic nerve as the yellow arrow shown.

One patient presented with an interesting clinical course worth highlighting given progression to secondary glaucoma within 3 weeks (Figure 2). Her mother noted a cloudy pupil in the left eye at birth. On the first examination at 3 months of age, the IOP was 11 mmHg in the right eye and 12 mmHg in the left eye. The slit-lamp examination showed a slightly larger pupil on the left associated with a white vascular band partially adherent to the inferior border of the pupil. Mild segmental iris bombé was found, but the central and peripheral ACD was still detectable with ACD of 3.5 corneal thickness. Dilated fundus examination was not possible due to the pupillary membrane, but the B-scan showed no optic nerve damage. Three weeks later, the examination was notable for increased IOP in the left eye (11.5 mmHg right eye and 23.8 mmHg left eye) and severe corneal edema. The central AC depth was 1.47 mm with peripheral iridocorneal touch. UBM demonstrated complete pupillary obstruction by the membrane adherent to the anterior lens capsule leading to iris bombé and peripheral anterior synechiae, consistent with type I CFPMSG. B-scan ultrasound demonstrated optic nerve cupping on the left and there was AL asymmetry (19.82 mm right eye and 21.49 mm left eye).

## Discussion

In this study, we summarize the clinical characteristics and propose a novel classification system of CFPMSG base on UBM images of 32 subjects: the largest series of this rare condition published to date. Overall, the clinical presentation (early-onset ocular hypertension, corneal enlargement and edema, AL elongation, and optic nerve cupping) was consistent with total pupillary blockage in the setting of the fibrotic membrane. UBM images confirmed obstruction of aqueous flow from the posterior chamber to the anterior chamber resulting in iris bombé and angle-closure glaucoma.

Secondary glaucoma is seen in CFPM often presents at an early age (12), and in our cohort, onset was between birth and 2 years of age with a median of 3 months. This early onset is consistent with prior reports of this condition, emphasizing the importance of early referral to a specialist. This early onset also supports prior suppositions that the pathophysiology likely occurs during embryogenesis. Unlike a similar condition, persistent pupillary membrane, which is thought to be a static condition, we found evidence of post-natal progression with CFPM. The normal anterior chamber angle continues to develop post-natally, which may explain the post-natal changes we observed in CFPM, such as the patient with no signs of glaucoma at 3 months of age who progressed to have iris bombe 1 month later. Enlargement and thickening of pupil-iris membrane led to pupil block and iris bombé, peripheral anterior synechia, and vision-threatening acute angle-closure glaucoma. Our study elucidated that the age at onset of glaucoma was around 1 month later than the age at presentation. In one of our cases (Figure 2), we also found the sudden elevation of IOP due to the seclusion of the pupil by the fibrovascular membrane. For this reason, close follow-up on patients with fibrotic pupillary membranes is recommended to monitor for the development of secondary glaucoma.

Given the rarity of this condition and a small number of reported cases, the incidence of secondary glaucoma is unknown. Due to our inclusion criteria, all of our CFPM cases had glaucoma. Of the cases of this condition previously published (Table 4), seven patients had glaucoma (4, 12–15). Given this and the secondary corneal edema associated with glaucoma, UBM is a useful adjunct in the identification and classification of anterior chamber involvement. As the UBM exam showed, there was no marked inflammatory activity. Therefore, we excluded the diagnosis of uveitis. UBM can be used to closely document and monitor abnormal attachments between the membrane and the iris, changes in ACD, membrane contracture, and malposition of the lens and ciliary processes (13).

**Table 4:** Summary of previous cases reports of the congenital fibrovascular pupillary membrane.

**No**.	**Author**	**Year**	**Cases**	**Secondary glaucoma**	**Age at glaucoma onset**	**IOP at glaucoma onset (mmHg)**
1	T. Avitabile et al.	2000	1	1	14 months	25
2	Richard M. Robb	2001	7	1	4.5 months	32
3	Gerhard W. Cibis	2004	9	3	7 days	45
					14 days	40
					2 months	40
4	Alexander Demidenko et al.	2009	1	1	3 days	45
5	Michiko Kandori et al.	2010	1	1	6 months	60

With UBM, we created a classification system based on the three phenotypes identified in our cohort. The majority of patients were in type I (“U” shape). The second most common was in type II (“Y” shape). The rarest and most severe type was type III (absent AC). Children with different UBM types of CFPMSG demonstrated different clinical characteristics. IOP was significantly higher in both types II and III patients than in type I patients and the ACD became shallower from type I to III, due to more severe manifestation of pupil block and iris bombé. In our cases, patients with type III were referred at a later age and presented with the most severe situation of secondary glaucoma. This partially demonstrated the severity increased with the duration of onset.

We found thickening of CCT in all three types of eyes, which may suggest the possibility of cornea decompensation even in a mild form of CFPMSG and close follow-up on corneal condition is important. Progression of CFPMSG from type I to III may occur with a more convex shape of iris and further shallowing of central anterior chamber subsequently leading to elevated IOP and optic nerve cupping. We also found the onset of secondary glaucoma at a younger age will be vulnerable to cornea enlargement and AL elongation. Therefore, it is necessary to have an early and close UBM follow-up for patients with CFPMSG to detect AC changes and disease progression.

Various treatment approaches for CFPM have been described. For patients without signs of secondary glaucoma, observation with or without adjuvant amblyopia treatment may be suitable for patients with normal IOP, deep anterior chamber, and sufficient pupillary opening. In cases with progressive growth and adhesion of the pupillary membrane which leads to microcoria and coretopia, timely membrane removal and separation, iridectomy, and anterior chamber reformation are required for preventing poor visual outcome (4, 16, 17). In eyes with secondary glaucoma, surgical excision of the membrane is the recommended treatment. For patients with iris bombé and extensive posterior synechiae, peripheral iridectomy, synechiolysis, goniosynechialysis, and pupilloplasty should all be considered dependent on the extent of the membrane (1, 4, 12). Since early detection facilitated early treatment, early surgical treatment is also recommended for preventing progressive and pathologic changes of CFPMSG.

There were several limitations to this study. First, given the rarity of this condition, only 32 cases are included, and another study is warranted to validate and confirm the clinical significance of the proposed classification system. Second, most of the enrolled subjects were younger than 1 year old, limiting visual acuity outcomes. Finally, the ages at which parents first noted symptoms were subjective and susceptible to recall bias and mild inaccuracy.

In conclusion, we describe the largest group of children with CFPMSG and presented a novel classification system based on UBM findings. Secondary glaucoma in this condition is due to pupillary block and angle-closure and requires surgical intervention. Unlike other congenital pupillary abnormalities, CFPMSG may be a progressive condition. UBM classification provides new insight into the diagnosis and monitoring of CFPMSG and may play a role in treatment planning.

## Data Availability Statement

The original contributions presented in the study are included in the article/supplementary material, further inquiries can be directed to the corresponding author/s.

## Ethics Statement

The studies involving human participants were reviewed and approved by Zhongshan Ophthalmic Center Institution Review and Ethics Board (2020KYPJ121). Written informed consent to participate in this study was provided by the participants' legal guardian/next of kin.

## Author Contributions

XL contributed to study decisions and clinical supervision. YTZ and LF were responsible for records review, data analysis, and manuscript writing. YMZ, JO, and YH contributed to data analysis and manuscript revision. SL, LC, XZ, YS, and PL contribute to ophthalmic examination and data record. All authors contributed to the article and approved the submitted version.

## Funding

This study was supported by the Sun Yat-sen University Clinical Research 5010 Program (2014016) by XL, the National Natural Science Foundation of China (81700858) by YTZ, and the Fundamental Research Funds of the State Key Laboratory of Ophthalmology by XL. The sponsor or funding organization had no role in the design or conduct of this research.

## Conflict of Interest

The authors declare that the research was conducted in the absence of any commercial or financial relationships that could be construed as a potential conflict of interest. The handling editor declared a shared affiliation with several of the authors YTZ, LF, YMZ, SL, LC, XZ, YS, PL, and XL at time of review.

## Publisher's Note

All claims expressed in this article are solely those of the authors and do not necessarily represent those of their affiliated organizations, or those of the publisher, the editors and the reviewers. Any product that may be evaluated in this article, or claim that may be made by its manufacturer, is not guaranteed or endorsed by the publisher.

## References

[1] Cibis GW Waeltermann JM Hurst E Tripathi RC Richardson W. Congenital pupillary-iris-lens membrane with goniodysgenesis. Ophthalmology. (1986) 93:847–52. 10.1016/S0161-6420(86)33659-5 3737130

[2] Cibis GW TR Tripathi BJ. Surgical removal of congenital pupillary-lris-lens membrane. Ophthalmic Surg Lasers. (1994) 259:580–3. 10.3928/1542-8877-19940901-06 7830997

[3] Lambert SR Buckley EG Lenhart PD Zhang Q Grossniklaus HE. Congenital fibrovascular pupillary membranes: clinical and histopathologic findings. Ophthalmology. (2012) 119:634–41. 10.1016/j.ophtha.2011.08.043 22197437 PMC3294086

[4] Cibis GW Walton DS. Congenital pupillary-iris-lens membrane with goniodysgenesis. J AAPOS. (2004) 8:378–83. 10.1016/j.jaapos.2004.04.010 15314601

[5] Lambert SR AL Taylor D. Congenital idiopathic microcoria. Am J Ophthalmol. (1988) 1065:590–4. 10.1016/0002-9394(88)90592-2 3189475

[6] Chu ER Taranath DA. Another case of congenital pupillary-iris-lens membrane with goniodysgenesis. J Pediatr Ophthalmol Strabismus. (2010) 47:e1–3. 10.3928/01913913-20100510-01 21214149

[7] Deshpande N SS Krishnadas SR. Pupillary-iris-lens membrane with goniodysgenesis: a case report. Indian J Ophthalmol. (2006) 544:275–6. 10.4103/0301-4738.27957 17090884

[8] Liang TW Zhang CY Bai DY Peng CX Bai XQ Wu Q. Clinical characteristics and treatment of congenital fibrovascular pupillary membranes. Zhonghua Yan Ke Za Zhi. (2018) 54:849–54. 10.3760/cma.j.issn.0412-4081.2018.11.010 30440157

[9] Temkar S Gupta S Sihota R Sharma R Angmo D Pujari A. Illuminated microcatheter circumferential trabeculotomy versus combined trabeculotomy-trabeculectomy for primary congenital glaucoma: a randomized controlled trial. Am J Ophthalmol. (2015) 159:490–7.e2. 10.1016/j.ajo.2014.12.001 25486542

[10] Tongu MTS Borges MJH Giovedi MRA Cohen R Almeida GV. Reliability of echographic examination for the study of optic nerve cupping. Arquivos Brasileiros de Oftalmologia. (1999) 62:265–8. 10.5935/0004-2749.19990010

[11] Roberto Sampaolesi RC. Ocular echometry in the diagnosis of congenital glaucoma. Arch ophthalmol. (1982) 100:574–7. 10.1001/archopht.1982.01030030576003 7073567

[12] RM R. Fibrous congenital iris membranes with pupillary distortion. Trans Am Ophthalmol Soc. (2001) 99:45–51. 11797319 PMC1359022

[13] Avitabile T Castiglione E Marano E Reibaldi M. Congenital pupillary-iris-lens membrane with goniodysgenesis: clinical history and ultrabiomicroscopic findings. J Pediatr Ophthalmol Strabismus. (2002) 394:248–50. 10.3928/0191-3913-20020701-17 12148562

[14] Demidenko A Jakobiec FA Hanna E Walton DS. Congenital pupillary-iris-lens membrane with goniodysgenesis: histopathologic findings in an enucleated eye. J Pediatr Ophthalmol Strabismus. (2010) 47:178–82. 10.3928/01913913-20100505-09 20507003

[15] Kandori M Saishin Y Kusaka S Shimojyo H Otori Y Tano Y. Pupilloplasty for congenital pupillary-iris-lens membrane with 25-gauge vitreous cutter. Acta Ophthalmol. (2010) 88:e289–90. 10.1111/j.1755-3768.2009.01698.x 19860772

[16] Wang Q Zhou F Chen W Chen J Ouyang Z Lin Z. A safe treatment for congenital fibrovascular pupillary membrane. Eur J Ophthalmol. (2020) 305:1143–8. 10.1177/1120672119859666 31256682

[17] Demidenko A JF Hanna E Walton DS. Congenital pupillary-iris-lens membrane with goniodysgenesis: a rare cause of glaucoma and vision loss. Int Ophthalmol Clin. (2009) 491:83–8. 10.1097/IIO.0b013e318192442b 19125067

